# Advancing health equity in the aftermath of COVID-19: Confronting intensifying racial disparities

**DOI:** 10.1016/j.isci.2024.110257

**Published:** 2024-06-12

**Authors:** Kristen R. Prentice, B. Adam Williams, Jane M. True, Charles H. Jones

**Affiliations:** 1 Rowan University, Glassboro, NJ, USA; 2 Pfizer Inc, 66 Hudson Boulevard, New York, NY 10001, USA

**Keywords:** health sciences, research methodology social sciences, social sciences

## Abstract

The COVID-19 pandemic has exposed and exacerbated the persistent racial and ethnic health disparities in the United States. The pandemic has also had profound spillover effects on other aspects of health and wellbeing, such as mental health, chronic diseases, education, and income, for marginalized groups. In this article, we provide a thorough analysis of the pandemic’s impact on racial and ethnic health disproportionalities, highlighting the multifaceted and interrelated factors that contribute to these inequities. We also argue for a renewed focus on health equity in healthcare policy and practice, emphasizing the need for systemic changes that address both the immediate and long-term consequences of these imbalances. We propose a framework for achieving health equity that involves creating equitable systems, care, and outcomes for all individuals, regardless of their race or ethnicity.

## Introduction

In a world grappling with the unprecedented challenges of coronavirus disease 2019 (COVID-19), a parallel crisis has emerged, casting a long shadow over our healthcare landscape—the intensification of racial and ethnic health disparities. This article aims to shed light on this critical issue, examining how the pandemic has not only exposed but also alarmingly exacerbated the deep-rooted inequities in health outcomes across racial and ethnic lines in the United States.

Health equity is the principle that every individual should have the opportunity to attain their highest level of health, regardless of their social or economic circumstances. Achieving health equity requires addressing the social and environmental determinants of health, which are the conditions in the environments where people are born, live, learn, work, and play that affect a wide range of health outcomes.^1^ Persistent racial and ethnic health disparities, a byproduct of historical and systemic inequalities, have been magnified under the lens of the COVID-19 pandemic. Health equity also requires eliminating health disparities, which are the differences in health outcomes, often observed along the lines of race, ethnicity, gender, age, disability, sexual orientation, and geographic location, among others, that are closely linked to social or economic disadvantage.^2^

The United States has a long history of racial and ethnic health inequities, which reflect the legacy of slavery, segregation, discrimination, and oppression that have shaped the social and economic status of racial and ethnic minority groups. These groups include Black or African American, Hispanic or Latinx, American Indian or Alaska Native, Asian, Native Hawaiian or Other Pacific Islander, and multiracial individuals, who collectively represent about 40% of the US population.^3^ These groups have historically faced barriers in accessing quality and culturally competent healthcare and often have increased exposure to social and environmental risk factors.^4^ These discrepancies not only affect individuals and families but also threaten the nation’s productivity and viability as, in 2018 alone, the cost attributed to racial and ethnic health disparities in the U.S. was estimated at $451 billion.

While health disparities exist in most, if not all, minoritized groups (e.g., race, ethnicity, LGBTQ+, disabled, immigrant, urban vs. rural, incarcerated, etc.), and all these populations have been impacted by the COVID-19 pandemic in unique ways, a detailed analysis of all minoritized groups is beyond the scope of this review. As such, discussion will be focused on racial and ethnic disparities in health, with a specific focus on Black and Hispanic Americans.

This article delves into the multifaceted nature of these disparities, highlighting the interplay of key social determinants of health, revealing that the COVID-19 pandemic has been not only a public health crisis but also a moment that calls for a critical examination of our healthcare system and its inadequacies in serving all segments of the population equitably. As we navigate through this analysis, we will explore how the pandemic has disproportionately impacted communities of color, not only in terms of immediate health outcomes but also in its broader spillover effects. The need for a renewed focus on health equity is more urgent than ever, demanding systemic changes to address both the immediate and long-term consequences of these disparities. Our goal is to propose a framework for achieving health equity that ensures equitable systems, care, and outcomes for individuals, regardless of their race or ethnicity.

## Social determinants of health

The first step in developing a framework for equitable outcome is understanding the impact of social determinants of health on marginalized groups that affect a wide range of health outcomes.^5^ Racial and ethnic minorities are more likely to experience disadvantages in social determinants which have been exacerbated by the pandemic and have contributed to the disparities in COVID-19 outcomes.^6^

### Housing

Housing is a key social determinant of health, as it affects the quality of life, the exposure to environmental hazards, and the ability to prevent and manage diseases. Racial and ethnic minorities are more likely to live in substandard or overcrowded housing, which can increase the risk of respiratory infections, asthma, lead poisoning, and injuries.^7^ They also experience housing instability, homelessness, or eviction at disproportionally higher rates compared with their White counterparts, which can affect their access to healthcare, education, and social services. For example, only 42% of Black Americans and only 46% of Hispanic Americans are homeowners, compared with 72% of White Americans.^8^ Additionally, despite making up only 13.4% of the US population in 2019, Black Americans made up almost 40% of the homelessness population. This is compared to the 47.7% of the homelessness population that is White, a demographic that made up 76.5% of the US population in 2019.^9^

The pandemic only increased these housing challenges for racial and ethnic minorities due to the loss of income, the lifting of the moratorium on evictions, and the risk of transmission in congregate settings. Two years following the lifting of the COVID-19 eviction moratoriums, more Americans were struggling to pay rent than ever before,^10^ with a disproportionate impact on racial minorities. For instance, 24% of Black renters surveyed in the US Census Bureau’s Household Pulse Survey were behind on rent payments, compared with 11% of White renters.^11^

### Education

Education affects income, employment, health literacy, and health behaviors of individuals and communities and is therefore considered one of the key social determinants of health. Academic success has long been considered a pathway for social and economic mobility, as it can provide opportunities for advancement and empowerment. However, racial and ethnic minorities are more likely to face disadvantages in education, such as lower access, quality, and outcomes, which impact their health outcomes and elevate their risk for diseases, including COVID-19.

Data on educational achievement reveal a concerning disparity. For example, the high school graduation rate in 2019 was 89% for White students, compared to 80% for Black students and 82% for Hispanic students.^12^ Additionally, of the students enrolled in undergraduate and other degree-granting postsecondary institutions in 2021, 52% were White, compared to 13% for Black students and 22% for Hispanic students.^13^ Furthermore, the 6-year college completion rate for White students was 64%, compared to 40% for Black students and 54% for Hispanic students.^14^

These educational disparities have direct and indirect impacts on income, employment, health literacy, and health behaviors. According to the Bureau of Labor Statistics, the median weekly earning for workers with a bachelor’s degree or higher was at least $1,608 in 2023, compared to $917 for workers with a high school diploma only.^15^ Moreover, the unemployment rate in 2022 for workers with a bachelor’s degree or higher was 2.2%, compared to 4.5% for workers with a high school diploma only.^16^

Academic disparities also have a direct link to health and wellbeing. As of 2018, the percentage of adults aged 25 years and older who reported poor health was 1.1% for those with a bachelor’s degree or higher, compared to 3.5% for those with a high school diploma only.^17^ This appears to correlate strongly with the breakdown by race, with 1.8% of White adults reporting poor health, compared with 2.9% of Black adults.^17^ Additionally, the percentage of adults aged 25 years and older who reported a limitation in daily activity due to a chronic disease, such as diabetes, heart disease, or cancer, was 9.0% for those with a bachelor’s degree or higher, compared to 18.9% for those with a high school diploma only.^17^

There is also a strong link between education attainment and behaviors with a strong impact on health outcomes, such as smoking, alcohol consumption, and maintaining an unhealthy diet.^18^ For example, the percentage of adults aged 25 years and older who reported smoking cigarettes in 2018 was 5.7% for those with a bachelor’s degree or higher, compared to 22.7% for those with a high school diploma only.^17^ Interestingly, there appears to be an inverse relationship between educational attainment and alcohol consumption, with 66% of those with a bachelor’s degree or higher reporting as regular alcohol consumers, compared with 47.2% of those with a high school diploma. However, individuals with fewer years of education more frequently report higher quantities during a single occasion and higher rates of alcohol-related health outcomes.^18^^,^^19^ Such behaviors contribute to increased risk from diseases, such as COVID-19, by compromising the immune system, making an individual more susceptible to infection.^20^

### Employment

Linked with education, employment is another key social determinant of health that affects income, access to health insurance, exposure to occupational hazards, and opportunities for social mobility.^21^ Racial and ethnic minorities are more likely to work in low-wage or essential sectors, such as healthcare, food service, transportation, and agriculture, which often have limited benefits, low job security, and elevated risk of exposure of transmissible diseases such as COVID-19. During the pandemic, for example, Hispanic Americans were disproportionately represented in frontline (58.3%) and blue-collar (51.4%) industries, compared with their White counterparts (38.7% and 41.2%, respectively).^22^ These industries were essential during the COVID-19 pandemic; however, due to the close proximity required, infection rates were high among people in these industries.^23^

Minority groups are also more likely to face discrimination, harassment, or exploitation in the workplace, which can affect their mental health, safety, and career advancement.^24^ According to a 2023 Pew Research Center poll, 41% of Black workers reported facing workplace discrimination due to their race, compared with 20% of Hispanic and 8% of White workers.^25^

### Healthcare access

Access to healthcare represents a pivotal social determinant of health, influencing not only the availability and affordability but also the quality of healthcare services, alongside the prevention and management of diseases. Racial and ethnic minorities are more likely to face barriers in accessing quality and culturally competent healthcare, such as lack of health insurance, lack of primary care providers, lack of transportation, language barriers, and discrimination.^26^ 63% of Black Americans, for example, stated in a 2021 Pew Research Center poll that less access to quality medical care in their area was a major reason for health inequities.^27^ They are also more likely to receive lower quality of care, such as fewer preventive screenings, less pain management, and less patient-centered communication.^28^ In the 2021 National Healthcare Quality and Disparities Report, 43% of Black Americans and 36% of Hispanic Americans were found to receive worse quality of care compared to White Americans.^29^

## Deepened racial health disparities in the COVID-19 era: An analytical overview

The effects of the COVID-19 pandemic on health equity are vast, complex, and difficult to fully quantify. Herein, we explore examples of the pandemic’s impact on racial and ethnic minority groups, covering four domains: health outcomes, economic implications, social determinants, and education. For this analysis, we highlight impacts on racial and ethnic groups, with a specific focus on Black and Hispanic populations. However, we acknowledge that all marginalized groups have been impacted in population-specific ways by the COVID-19 pandemic and the trends discussed below may not apply to all racial or ethnic groups.

### Disparities in COVID-19 infection, hospitalization, and mortality

The COVID-19 pandemic has had a disproportionate impact on racial and ethnic minority communities, leading to higher rates of infection, hospitalization, and mortality. In 2021, one year into the pandemic, the age-adjusted COVID-19 mortality rate for Black Americans was 1.9 times that of White Americans, while the rate for Hispanic Americans was 2.3 times that of White Americans.^30^^,^^31^ Similarly, the age-adjusted COVID-19 hospitalization rate for Black Americans was 2.8 times that of White Americans, while the rate for Hispanic Americans was 3.0 times that of White Americans.^30^^,^^31^ These disparities are even more pronounced when considering the younger age distribution of racial and ethnic minorities, as they account for a larger share of COVID-19 deaths and hospitalizations among those under 65 years of age. In 2021, Black Americans represented 13.6% of the US population^32^^,^^33^ but experienced 28.7% of COVID-19 deaths and 33.9% of COVID-19 hospitalizations among those under 65 years of age.^34^ Similarly, Hispanic Americans represented 19.1% of the US population,^32^^,^^35^ but 28.9% of COVID-19 deaths and 32.5% of COVID-19 hospitalizations among those under 65 years of age.^34^

These inequities reflect the underlying health disparities that have made racial and ethnic minorities more vulnerable to the pandemic, including higher prevalence of comorbidities, lower access to quality healthcare, and greater exposure to occupational and environmental risks. Racial and ethnic minorities are more likely to suffer from chronic conditions, such as diabetes, hypertension, cardiovascular (CV) disease, obesity, and asthma, which increase the risk of severe illness and death from COVID-19. As of 2018, the age-adjusted prevalence of diabetes among Black Americans was 11.7% and among Hispanic Americans was 12.5%, compared to 7.5% among White Americans,^36^ and diabetes was a contributing factor for 38% of COVID-19 deaths among Black Americans, as well as 36% of COVID-19 deaths among Hispanic Americans, compared to 29% of COVID-19 deaths among White Americans. Additionally, racial and ethnic minorities are more likely to face barriers in accessing quality and culturally competent healthcare, such as lack of health insurance, lack of primary care providers, lack of transportation, language barriers, and discrimination.^37^ For example, 10% of the Black American and 18.0% of the Hispanic American populations were uninsured in 2022, compared with 6.6% of White Americans.^38^ Additionally, racial and ethnic minorities are more likely to work in low-wage or essential sectors, such as healthcare, food service, transportation, and agriculture,^39^ which increase the risk of exposure and transmission of COVID-19.^40^ They are also more likely to live in substandard or overcrowded housing, situations that have been linked to increased spread and mortality associated with COVID-19.^41^ Additionally, marginalized groups in the United States are more likely to experience higher levels of exposure to air pollution. The 2022 State of the Air report found that people of color were 61% more likely to live in counties with a failing grade for at least one pollutant than White Americans.^42^ This is particularly troubling as pollution has been found to worsen respiratory conditions that can increase the susceptibility to severe COVID-19-related outcomes.^43^

The health outcome disparities experienced by racial and ethnic minorities are the result of a complex interplay of biological, behavioral, social, and environmental factors, which are shaped by the historical and contemporary patterns of racial discrimination and oppression in the US society. These factors operate at multiple levels, from the individual to the structural, and interact in synergistic and cumulative ways, creating a cycle of disadvantage and vulnerability for racial and ethnic minorities.^44^ At the individual level, racial and ethnic minorities may experience higher levels of stress, which can affect their immune system, their health behaviors, and their mental health.^45^ At the interpersonal level, racial and ethnic minorities may encounter bias, prejudice, or stereotyping from healthcare providers, which can affect their quality of care, their trust in the healthcare system, and their adherence to treatment.^46^ At the institutional level, racial and ethnic minorities may face limited access to healthcare resources, such as testing, treatment, or vaccination. At the structural level, racial and ethnic minorities may experience unequal opportunities and outcomes in education, employment, income, and housing, which can affect their social and economic status, their living conditions, and their exposure to risk factors.^47^ These factors are not mutually exclusive, but rather mutually reinforcing, creating a web of causation and a gradient of risk for racial and ethnic minorities. At every level, these factors impact the ability of marginalized groups to prevent and manage COVID-19, increasing their susceptibility and worsening outcomes.

The impact of these disparities can be seen in the age-adjusted rates of COVID-19 cases, hospitalizations, and deaths by race and ethnicity early in the pandemic (Table 1). Black Americans and Hispanic Americans experienced the highest rates and ratios of disparate COVID-19 outcomes, followed by Native Americans and Pacific Islanders, while Asian Americans experienced the lowest rates and ratios of disparate COVID-19 outcomes, compared to White Americans.

**Table 1:** Age-adjusted rates of COVID-19 cases, hospitalizations, and deaths by race and ethnicity in the US in December 2020

Race or Ethnicity	COVID-19 Cases		COVID-19 Hospitalizations		COVID-19 Deaths	
	per 100,000	Ratio to White Americans	per 100,000	Ratio to White Americans	per 100,000	Ratio to White Americans
White	5,094	1.0	241	1.0	97	1.0
Black	6,282	1.2	1,132	4.7	236	2.4
Hispanic	9,403	1.8	1,111	4.6	203	2.1
Native American	8,197	1.6	1,055	4.4	217	2.2
Pacific Islander	7,980	1.6	1,022	4.2	180	1.9
Asian	2,925	0.6	186	0.8	54	0.6

### Employment instability

Economic impacts from the COVID-19 pandemic lead to higher rates of unemployment, income loss, and financial insecurity among racial and ethnic minorities compared to White Americans.^48^ The pandemic triggered a severe economic recession, lasting only two months,^49^ which disproportionately affected the sectors and occupations where racial and ethnic minorities are overrepresented, such as hospitality, leisure, retail, and personal services. By the end of 2020, marginalized populations represented a larger proportion of unemployed adults, with unemployment rates of 11.4% for Black Americans and 10.4% for Hispanic Americans, compared with the unemployment rate 7.3% for White Americans.^50^ This is supported by a survey conducted by the Pew Research Center which found that 43% of Black adults and 53% of Hispanic adults reported that they or someone in their household had lost a job or taken a pay cut due to the pandemic, compared to 38% of White adults.^48^ Furthermore, 43% of Black adults and 37% of Hispanic adults reported that they had trouble paying their bills in the past year, compared to 18% of White adults.^48^ The financial strain from such income loss can lead to difficulties in affording healthcare, resulting in delayed or foregone medical care, which can worsen health conditions.

The impact of these economic disparities, which were not only highlighted by but may have also been exacerbated by the pandemic, has worsened pre-existing health inequities, underscoring the critical need for targeted interventions to support these vulnerable populations economically and thus protect their health.

### Insurance and healthcare access

The elevated rate of unemployment among minority groups also translated into a loss of employer-sponsored health insurance and increased economic vulnerability; although this was mitigated by the disproportionate dependence of these groups on government-sponsored programs, such as Medicaid. As of 2022, approximately 74% of White Americans had employer-sponsored or private health insurance, compared to 51% of Black Americans and 49% of Hispanic Americans.^38^ Additionally, only approximately 20% of White Americans had Medicaid coverage, compared to 39% of Black Americans and 34% of Hispanic Americans.^38^ The loss of employment or income due to a pandemic can result in losing eligibility for these sources of coverage, or in facing difficulties in affording premiums, deductibles, or copayments.^51^ This, in turn, can lead to reduced access to healthcare services, especially for preventive and chronic care, and increased financial burden from medical bills. Interestingly, the increase in financial burdens brought about by the COVID-19 pandemic did not increase the rate at which Black and Hispanic Americans avoided healthcare due to cost, according to a 2023 Commonwealth Fund report. In fact this decreased by 3.1% and 4.5% points for Black and Hispanic adults, respectively, from 2019 to 2021.^52^ However, between 14% and 18% of these populations were reporting cost as a reason for avoiding care in 2021, significantly more than their White American counterparts at 9.5%. More Black and Hispanic Americans also faced difficulties in paying for medical bills in 2021 (15.8% and 12.8%, respectively) than White Americans (9.4%).^53^

The COVID-19 pandemic also created barriers to healthcare access, including access to COVID tests and vaccinations, for racial and ethnic minorities due to the disruption of routine services, the diversion of resources, the fear of exposure, and rise in mistrust of the healthcare system.^54^ For example, attempts to regulate the demand for COVID-19 testing in places such as New York, which necessitated prescriptions from physicians for testing, inadvertently established structural obstacles for minorities who frequently lack access to primary healthcare.^55^ Successive initiatives for “drive-thru” testing, while intended to broaden access, still required car ownership, thereby marginalizing those reliant on public transportation or without a physician. These measures disproportionately impacted racial and ethnic minorities, intensifying access problems with the steep expenses of emergency room visits for individuals without alternative testing options.

The disparities highlighted by the COVID-19 pandemic underscore the urgent need for systemic changes to address insurance and healthcare access inequities faced by minority communities. It has become increasingly important to ensure access to affordable healthcare and support mechanisms. By acknowledging the unique barriers these communities face, including mistrust of the healthcare system and disproportionate financial burdens, we can begin to implement solutions that not only address the immediate impacts of the pandemic but also work toward long-term health equity. Strengthening public programs, expanding healthcare access, and fostering trust within these communities are essential steps in mitigating the pandemic’s lasting effects and moving toward a healthier, more inclusive future.

### Food insecurity and nutrition

The pandemic also increased the risk of food insecurity among racial and ethnic minorities, who are more likely to experience hunger or lack of access to adequate and nutritious food than White Americans. Even before the pandemic, 19.1% of Black households and 15.6% of Hispanic households were food insecure, compared to 7.9% of White households.^56^ Food insecurity can have negative health consequences, such as increased risk of obesity, diabetes, hypertension, and CV disease, and impaired cognitive and emotional development among children,^57^ trends that are already observed in marginalized groups.

The COVID-19 pandemic exacerbated food insecurity due to the loss of income, the disruption of school meals, the closure of food pantries, and the increased demand for food assistance. According to the US Department of Agriculture (USDA), in 2020, 21.7% of Black households and 17.2% of Hispanic households experienced food insecurity, compared to 7.1% of White households.^58^ During the height of the pandemic in 2020, 22% of Black households and 19% of Hispanic households with children reported that their children were not eating enough, compared to 9% of White households with children.^59^ Additionally, there was a 55% increase in the number of individuals using food banks from 2019 to 2020, with communities of color being the most reliant on these services.^60^ Food assistance programs have become a lifeline for many, yet they are often underfunded and unable to meet the increased demand, leaving many families without adequate support.

These alarming statistics highlight the urgent need for comprehensive strategies to address food insecurity, which is both a cause and a consequence of health disparities. Ensuring access to nutritious food is not just a matter of economic policy but also essential for the prevention of disease and the promotion of health equity.

### Mental health strain

Counterintuitively, minoritized groups experience mental health problems at the same rate, or even at lower rates, than their White counterparts. However, the consequences for those that do experience mental health problems are often more severe and long lasting,^61^ a trend that was only exacerbated by the COVID-19 pandemic. As of 2020, 17% of Black adults and 19% of Hispanic adults reported having any mental illness, compared to 26% of White adults.^62^ However, racial and ethnic minorities are less likely to receive mental health services, due to factors such as stigma, cost, lack of insurance, lack of availability, and lack of cultural competence.^62^ For adults with moderate or severe anxiety and/or depression, 65% of White adults surveyed by the Kaiser Family Foundation (KFF) in 2019 received mental health services, compared to 47% of Black adults and 60% of Hispanic adults.^62^

The pandemic’s psychological toll is immense, with heightened levels of stress, anxiety, and depression. While these conditions affect the entire population, racial and ethnic minorities often have less access to mental health services. The spillover effects on mental health can exacerbate existing health disparities, as untreated mental health conditions often lead to worsened overall health outcomes. As of 2021, 30% of Black adults and 26% of Hispanic adults surveyed by the KFF reported that the pandemic had a major negative impact on their mental health, compared to 14% of White adults.^62^ In minoritized communities, there has been a notable increase in anxiety and depression symptoms among these populations.^63^

These stressors, if prolonged, can have detrimental effects on physical health, such as increasing the risk for chronic conditions like heart disease and diabetes. The mental health strain induced by the pandemic underscores a critical area of health disparities that demands urgent attention. It is essential to improve access to mental health services and provide targeted support to those communities most affected by these unprecedented stressors.

## Spillover effects from the pandemic

The spillover effects from the COVID-19 pandemic extend well beyond the immediate health implications, significantly shaping public attitudes and behaviors, particularly in the realm of vaccinations. This complex interplay between public health responses to the pandemic and established health practices has manifested in various forms, significantly impacting vaccine uptake and healthcare services, while exacerbating existing disparities.

### Wider repercussions on vaccine hesitancy

The attitudes toward the COVID-19 vaccine have been a critical factor in shaping public health responses and outcomes during and after the pandemic, revealing deep-seated disparities in trust and acceptance across different communities. Particularly among racial and ethnic minorities, such as Black and Hispanic Americans, skepticism and hesitancy toward the vaccine reflect not only concerns about its safety and efficacy but also broader issues of historical and systemic mistrust in the healthcare system.^64^^,^^65^ For example, only 17% of Black Americans said they would definitely receive the COVID-19 vaccine in a 2020 KFF report, 20% points lower than both Hispanic and White Adults (37%).^66^ 39% of those saying they would not get the vaccine cited distrust in the healthcare system, government, or vaccines in general as the main reason why.

The impact of the pandemic on vaccine trust is not limited to the COVID-19 vaccine. One of the most striking examples of these spillover effects is the cognitive process known as “belief generalization,” which carries substantial implications for health disparities among racial and ethnic minorities, such as Black and Hispanic Americans, within the framework of the COVID-19 pandemic.^67^^,^^68^ This psychological mechanism, wherein individuals transpose beliefs from one realm (such as views pertaining to COVID-19 vaccines) to another (on to influenza vaccines), is shaped by emotions, societal norms, media exposure, and personal experiences. In the context of the pandemic, belief generalization can explain why some people who are hesitant or resistant to COVID-19 vaccines may also avoid or reject other vaccines, such as influenza or measles, even if they have different safety profiles, efficacy rates, or disease risks. Conversely, belief generalization can also explain why some people who are motivated or persuaded to get COVID-19 vaccines may also seek or accept other vaccines, such as those for influenza or human papillomavirus (HPV) infection, even if they have different benefits, costs, or availability. For Black and Hispanic communities, a historical skepticism of the healthcare system, propelled by systemic racism and past injustices, intensifies hesitancy toward not only COVID-19 vaccines but also other vital vaccinations, like those for influenza.

Recent data suggest that vaccine hesitancy in general is more prevalent among Black and Hispanic populations as compared to their White counterparts, a disparity deeply entrenched in historical and enduring healthcare inequities. A 2023 study conducted by Zhang et al. found that 60% of Black and 48% of Hispanic Americans surveyed were vaccine hesitant, compared with 35% or White Americans.^69^ While vaccine hesitancy was high for both demographics, this did not translate to low COVID-19 and influenza vaccine uptake among Hispanic Americans, as the non-recipient rates were similar to those of White Americans (10%–16% and 31%–32%, respectively). However, there was a significant spillover effect from vaccine hesitancy observed among Black Americans, who had nearly double the rate of non-receipt for both vaccines (39% and 59%, respectively). The differences were attributed to differences in healthcare provider trust, and other systemic factors resulting from the historical roots of racism and segregation.

The spillover effects of COVID-19 vaccine attitudes on perceptions of other vaccines highlight that tackling these disparities demands a multifaceted strategy that not only concentrates on augmenting vaccine access and affordability but also fosters trust in the healthcare system through transparency, education, and community involvement. By comprehending and addressing the root causes of vaccine hesitancy in these communities, public health officials can strive to bridge the gap in health disparities and ensure equitable health outcomes.

### Interrupted education and health literacy

The educational impact of the pandemic also deserves attention. School closures and the shift to remote learning have disrupted traditional educational pathways, with disproportionate effects on minority and low-income students. This disruption has affected not only academic learning but also access to school-based health services, including vaccinations and mental health support.

The digital divide has exacerbated these educational disparities, with many students in minority communities lacking access to reliable internet and devices necessary for effective remote learning. Even before the pandemic, a 2018 Pew Research Center poll found that 25% of Black teens and 17% or Hispanic teens reported that they were unable to complete homework at home due to a lack of reliable computer or internet connection, compared with 13 percent of White teens.^70^

The pandemic further increased these educational challenges for racial and ethnic minorities, due to the lack of access to technology, health services, quality instruction, and social and emotional support. According to a 2020 report by Common Sense Media, 30% of Black and 26% of Hispanic students lived in households without adequate internet access needed for online learning, compared with 18% of White students.^71^ This impact can be seen in student’s participation in virtual learning during the pandemic. According to a survey by the RAND Corporation, as of May 2020, 42% of teachers in schools with high percentages of minority students reported that their students were not regularly participating in remote learning during the pandemic, compared to 18% of teachers in schools with low percentages of minority students.^72^ This shift has raised concerns about widening educational disparities and the long-term impacts on student learning and development. Educational institutions, traditionally central in promoting health literacy and wellbeing, have struggled to maintain these roles amid the pandemic, highlighting the critical need for resilient and adaptive educational systems.

### Long-term implications

The profound and far-reaching racial disparities exacerbated by the COVID-19 pandemic in the US highlight the critical need for comprehensive strategies to effectively address these inequities. The pandemic has illuminated and exacerbated the structural inequities present in healthcare, education, and employment, establishing the groundwork for lasting impacts on health outcomes and economic stability among racial and ethnic minority communities.

An immediate concern is the effects of the disruption of preventive care and chronic disease management during the pandemic, particularly within Hispanic and Black communities, where conditions such as CV disease, cancer, and diabetes are disproportionately prevalent. The limited access to healthcare services, exacerbated by heightened economic stress, has the potential to create a cycle of ill health and economic hardship.

Furthermore, the pandemic’s interference with routine healthcare services has exposed the entrenched inequities within the US healthcare system. Minority communities, already confronting barriers to accessing care, have experienced worsened disparities, accentuating the systemic nature of these challenges. The secondary effects of such disruptions are likely to emerge as more severe health issues in the future, further deepening health disparities.

In order to address these enduring implications, a comprehensive approach is necessitated—one that not only concentrates on the direct health impacts of the pandemic but also confronts the underlying structural inequities in healthcare, education, and employment. Efforts must be diverse, aiming to enhance access to quality healthcare, improve economic opportunities, and ensure equitable educational resources. It is only through such unified efforts that the US can aspire to mitigate the enduring impact of COVID-19 on racial disparities, fostering a more equitable and healthy future for all communities.

## A call to action: Transforming the healthcare landscape for equity and justice

**Figure 1:**
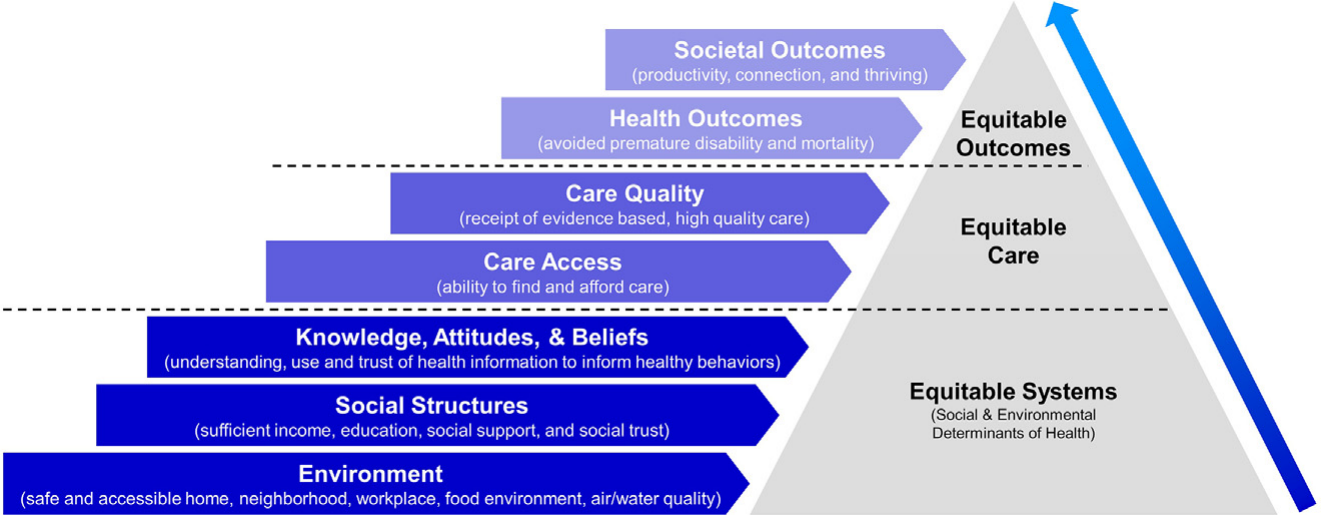
A framework for equitable health outcomes This figure illustrates the multifaceted approach needed to achieve health equity, encompassing several layers from environmental conditions to societal outcomes. This framework supports access to and the quality of care, leading to direct health outcomes and, ultimately, broader societal benefits like increased productivity and thriving communities. Each level underscores a critical component of health equity, emphasizing that interventions must target multiple aspects of health determinants to foster an equitable healthcare system. This model serves as a framework for policymakers, healthcare providers, and communities aiming to bridge health disparities and promote a healthier, more equitable society.

The urgent need for systemic changes in healthcare policy to tackle deepened health disparities has been underscored by the COVID-19 pandemic. Achieving health equity requires a concerted effort across various levels of policy and practice, focusing on equitable systems, care, and outcomes (Figure 1). This involves ensuring that all individuals have access to quality healthcare, regardless of their socioeconomic status, and addressing the broader social determinants of health.

### Crafting equitable systems

The pursuit of health equity necessitates the construction of healthcare systems that are inherently just and unbiased. This involves comprehensive policy reforms to ensure equitable distribution of healthcare resources. Policies must prioritize the expansion of healthcare coverage, targeting underserved and minority communities. An emphasis on improving healthcare delivery in these areas is crucial, as is the investment in community health initiatives that are culturally sensitive and responsive to the specific needs of these populations.

### Addressing social determinants of health

Equity extends beyond healthcare access; it encompasses the broader social determinants of health. Efforts must focus on enhancing living conditions, amplifying employment opportunities, fostering income equality, and ensuring equitable educational access. These determinants are pivotal in shaping health outcomes and are integral to achieving true health equity.

### Combating systemic racism in healthcare

A critical aspect of this transformation is addressing the insidious role of systemic racism within the healthcare sector. This requires a proactive approach to increase awareness about racial health disparities, coupled with a commitment to collecting and analyzing data that can inform more equitable policies and practices. It is only through recognizing and actively opposing these ingrained biases that there exists a possibility of dismantling the barriers to equitable healthcare.

## Conclusion: The imperative of urgency

At this crucial juncture, the COVID-19 pandemic serves not just as a reminder but also as a stark warning of the deep-seated inequities that pervade our healthcare system. The disparities laid bare by this crisis are not just statistics; they represent real lives, real suffering, and a legacy of injustice that demands immediate and resolute action.

The path toward health equity is challenging, yet imperative. It calls for a collective commitment to systemic change, a relentless pursuit of justice, and an unwavering dedication to the principle that health is a fundamental human right, not a privilege. Action must be taken with urgency and determination, for the cost of inaction is measured in human lives. In striving for a healthier, more equitable future, it is imperative to remember that the time to act is not tomorrow, but today.
